# Case Report: Kearns Sayre Syndrome Complicated With Postpartum Cardiac Failure

**DOI:** 10.3389/fmed.2022.906112

**Published:** 2022-06-20

**Authors:** Caixia Han, Zongyang Jia, Guangcai Zhao, Wenhui Chen, Yue Hu, Haiying Liu

**Affiliations:** Department of Obstetrics and Gynecology, Cheeloo College of Medicine, Qilu Hospital (Qingdao), Shandong University, Qingdao, China

**Keywords:** Kearns Sayre Syndrome, pregnancy, postpartum, mitochondrial, cardiac failure, case report

## Abstract

Kearns Sayre Syndrome (KSS) is a rare mitochondrial disease characterized by a primary dysfunction of the mitochondrial respiratory chain. Cardiac involvement is a poor prognostic factor of KSS. Pregnancy and delivery in a KSS patient with cardiac involvement is uncommon, and strategies for the supervision and management of this group remain unclear. Herein, we report and discuss pregnancy and delivery complicated with acute cardiopulmonary failure in a woman with KSS.

## Introduction

Mitochondrial disease (MD) refers to a clinically heterogeneous group of disorders that arise from dysfunction of the mitochondrial respiratory chain due to a genetic abnormality in nuclear or mitochondrial DNA (mtDNA). The conservative estimate for the prevalence of MD is 11.5:100,000 (1). Due to the complete dependence on oxidative metabolism, cardiac involvement in MD may occur as a major clinical complication or part of a multisystemic disease. The mortality in MD patients with cardiac involvement is much higher than in patients without cardiac involvement (71 vs. 26%) (2). Kearns Sayre syndrome (KSS), a rare and severe form of MD, is initially characterized by a clinical trial of progressive external ophthalmoplegia (PEO), pigmentary retinal degeneration, and onset before age 20. In contrast to the other MDs, cardiac involvement, as a contributing factor to the diagnosis, is common and often a major determinant of outcome in KSS. Cardiac manifestations occur in up to 57% of KSS patients, and sudden cardiac death (SCD) has been reported in up to 20% of KSS patients (3). There are a few reports of pregnancy and uneventful delivery in women affected with MD. However, data about pregnancy and delivery in KSS with cardiac involvement are rare. Potential predictors of adverse cardiac events during the gestational and postpartum periods for KSS with cardiac involvement are unclear.

## Case Description

**Figure 1:**
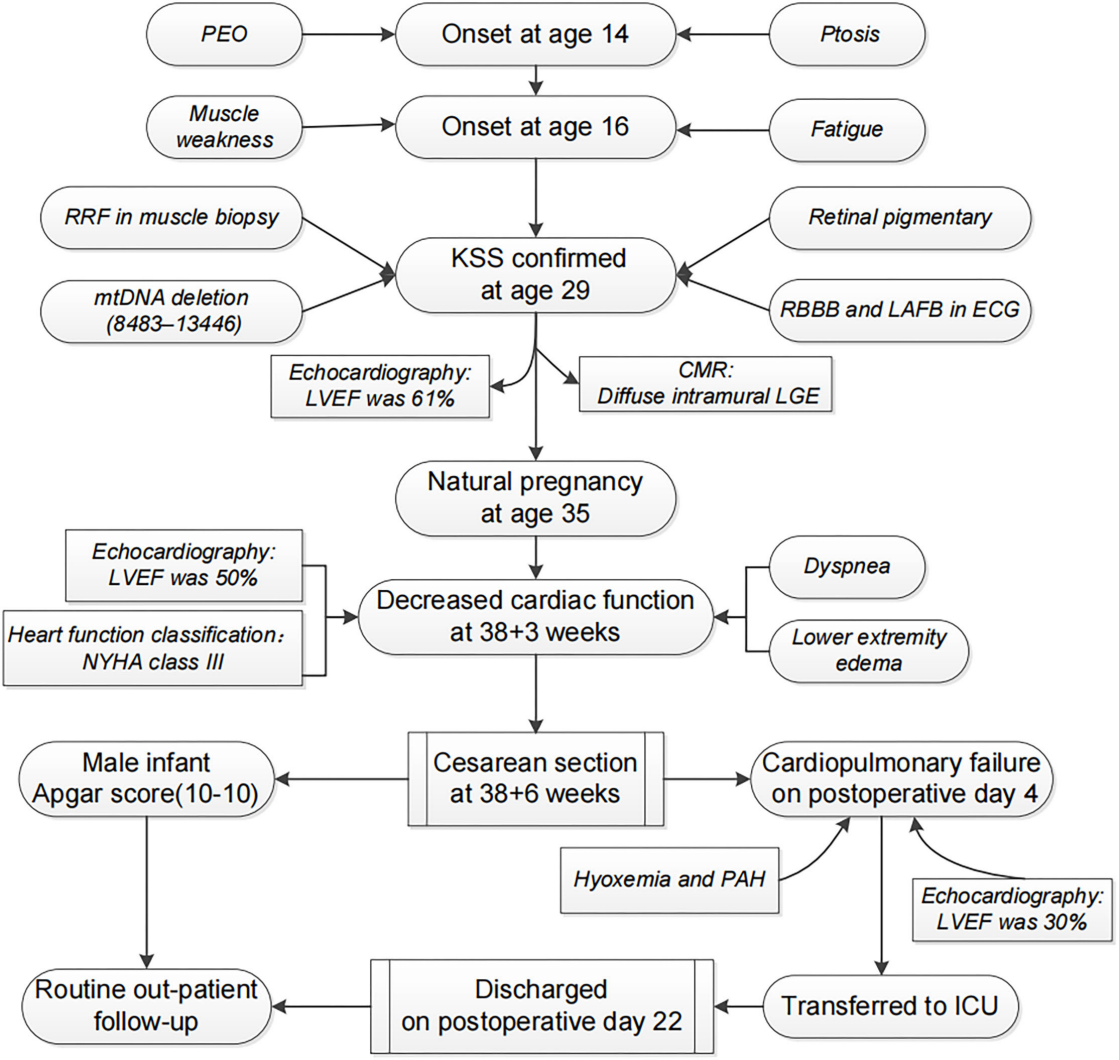
A schematic diagram of the progression of disease in the current patient.

A 35-year-old woman developed bilateral ptosis and PEO at age 14. Progressive muscle weakness and fatigue were noted from age 16. The diagnosis of KSS was confirmed at age 29 by the above symptoms and subsequent laboratory findings, and the process is summarized in Figure 1. She was the only child of her nonconsanguineous parents. There was no family history of such conditions. No mtDNA deletion in her parents' peripheral blood was found. Her menarche was at age 14, and her menstrual cycle was regular. Her cognitive and physical development were normal. Previous routine screening of the main endocrine organs,

including the pancreas, thyroid, parathyroid, and ovary, demonstrated no associated endocrine dysfunction. Her medications included multivitamins, idebenone, and coenzyme Q10. Her condition showed no serious progression since the beginning of supportive therapy. She had no new occurrence of other symptoms, such as dysphagia, hearing loss, syncope, or dyspnoea.

**Figure 2:**
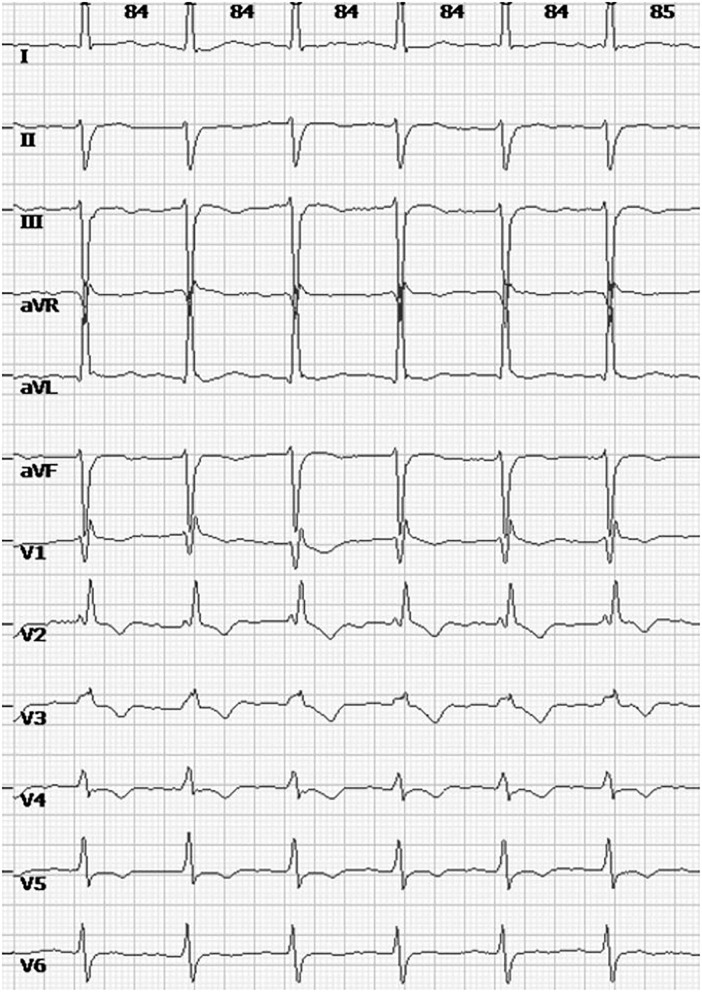
12-lead ECG showing RBBB and LAFB.

The patient had a first and natural pregnancy at age 35. A systematic cardiac assessment was performed 2 months before her pregnancy, while an electrocardiogram (ECG) showed a preexisting complete right bundle branch block (RBBB) and left anterior fascicular block (LAFB) (Figure 2). Echocardiography showed normal ventricular size and function without valvular abnormalities, and the left ventricular ejection fraction (LVEF) was 61%. Cardiovascular magnetic resonance imaging (CMR) showed diffuse intramural late gadolinium enhancement (LGE) in the left ventricular inferolateral walls. There were no restrictions on her routine activities during the first and second trimesters. Echocardiography performed at 28 weeks also showed no abnormal findings. Antenatal inspections were uneventful except for a finding of gestational diabetes mellitus (GDM). She maintained an acceptable blood glucose level with a diabetic diet and proper physical activity.

The patient was referred to our hospital at 38+3 weeks with complaints of lower extremity oedema and progressive dyspnoea after routine activity. Physical examination revealed a good nutritional status (height 162 cm, weight 58 kg, BMI 22 kg/m^2^), bilateral palpebral ptosis, external ophthalmoplegia, and proximal muscle weakness. Tendon reflexes of limbs had disappeared. The oedema of the lower extremity was up to the level of the knee. Laboratory results showed normal levels of hemoglobin, brain natriuretic peptide (BNP), D-dimer, and lactic acid. Arterial blood gas analysis showed no obvious hypoxemia, pH, bicarbonate, and PCO2 were within normal limits. Cardiac assessment presented no obvious ECG changes compared with the previous results. At the same time, a reduced LVEF (50%) and mild dilation of the left ventricle were found by echocardiography. She was in NYHA class III. After a comprehensive evaluation of her condition by a multidisciplinary team, a cesarean section (CS) under combined spinal-epidural anesthesia was performed at 38+6 weeks. A 3.65 kg male infant was delivered with Apgar scores of 10-10 at 1 and 5 minutes after delivery. The surgery was uneventful, and the intraoperative blood loss was ~200 ml.

**Figure 3:**
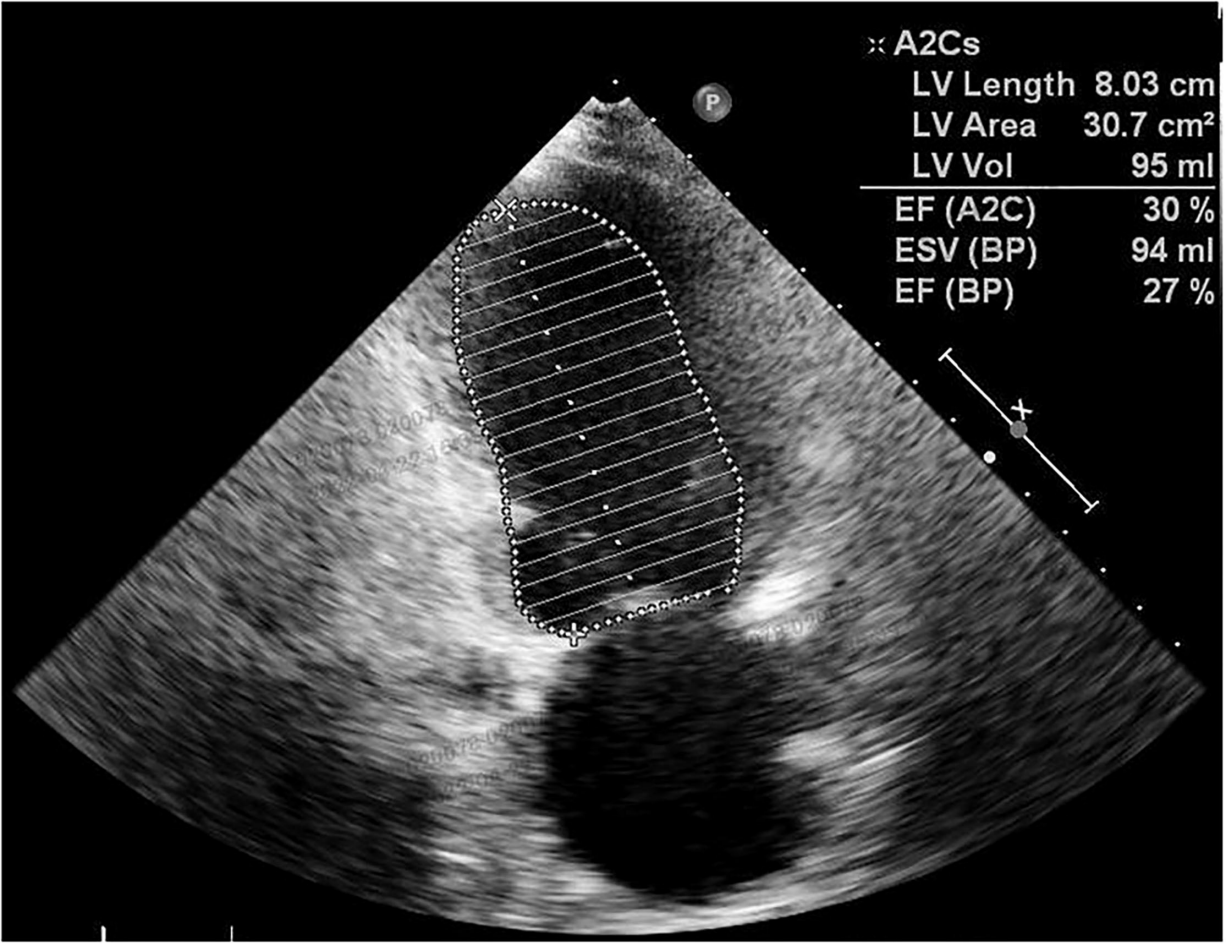
Echocardiography performed on a postoperative Day 4 showed a remarkable decrease in LVEF.

Postpartum therapy consisted of intravenous anti-infective therapy, diuretics, and cardiac drugs, the first three postoperative days were uneventful. However, cardiopulmonary function suffered a sudden deterioration on postoperative Day 4. The patient complained of dyspnoea. Even with a high-flow oxygen supply, the arterial blood gas analysis showed that the oxyhemoglobin saturation was only 70%, artery oxygen pressure (PaO_2_) was 56 mmHg, and carbon dioxide pressure (PaCO_2_) was 87 mmHg. The BNP value reached 1234 pg/ml. ECG showed a new first-degree atrioventricular (AV) block with preexisting RBBB and LAFB. Echocardiography showed decreasing systolic function of the left ventricle (LVEF was 30%) (Figure 3) and pulmonary arterial hypertension (PAH: 36 mmHg). Given all the above information, the patient was diagnosed with acute cardiopulmonary failure. She received mechanical ventilation with endotracheal intubation and was transferred to the intensive care unit (ICU). After 5 days of comprehensive therapy, the patient gradually recovered from the cardiopulmonary dysfunction and was then transferred to the department of neurology for specialist treatment. Echocardiography performed 18 days after delivery showed a mild dilation and regional wall motion abnormality of the left ventricle (LVEF was 55%). She was discharged on postoperative Day 22 in a stable condition. The related laboratory data during hospitalization are summarized in Table 1.

**Table 1:** Laboratory data of the patient during her hospitalization.

**Indicator**	**Preoperative period (the day before CS)**	**Postoperative Day 4 (onset of AHF)**	**Postoperative day 18 (before discharge)**	**Normal range**	**Unit**
WBC	4.63	9.88	4.77	3.5 ~ 9.5	× 10^9^/L
HGB	12.4	12.1	13.1	11.5 ~ 15.0	g/dL
PLT	167	147	351	125 ~ 350	× 10^9^/L
CRP	/	155	6.5	0 ~ 8	mg/L
PT	9.90	10.30	11.90	8.8 ~ 13.8	s
APTT	31.10	30.00	35.80	26 ~ 42	s
FIB	4.21	5.11	4.15	2.0 ~ 4.0	g/L
D-dimer	3.40	3.87	1.94	0.00 ~ 0.55	mg/L
ALT	21	23	22	7 ~ 40	U/L
AST	29	30	17	13 ~ 35	U/L
ALB	35.1	34.2	45.8	35 ~ 55	g/L
Glu	4.10 (fasting)	10.40 (random)	4.84 (fasting)	3.9 ~ 6.1 (fasting) <11.1 (random)	mmol/L
SCr	37	29	41	44 ~ 80	umol/L
BUN	3.7	4.88	5.55	2.3 ~ 7.8	mmol/L
Serum Na^+^	137	142	142	137 ~ 147	mmol/L
Serum K^+^	4.26	3.9	4.28	3.5 ~ 5.3	mmol/L
BNP	90	1234	150	<100	pg/mL
TnI	0.04	0.08	0.02	0.00 ~ 0.06	ng/mL
pH*	7.36	7.24	7.4	7.35 ~ 7.45	/
${\text{PaCO}}_{2}^{*}$	80	56	81	80 ~ 100	mmHg
${\text{PaCO}}_{2}^{*}$	45	87	40	35 ~ 45	mmHg
BLA*	1.5	2.40	1.2	0.5-1.8	mmol/L

*WBC, white blood cell; HGB, hemoglobin; PLT, platelets; CRP, C-reactive protein; PT, prothrombin time; APTT, activated partial thromboplastin time; FIB, fibrinogen; ALT, alanine aminotransferase; AST, aspartate aminotransferase; ALB, albumin; Glu, glucose; SCr, serum creatinine; BUN, blood urea nitrogen; TnI, troponin I; BLA, blood lactic acid*.

* *data from blood gas analysis*.

Over more than 5 years of outpatient follow-up, the patient was in a stable condition except for a slight progression of muscle weakness and ophthalmoplegia. The increased glucose level gradually returned to normal within 2 months after delivery and without recurrence. Compared with the previous results at discharge, echocardiography performed 6 months before the paper was submitted showed no remarkable changes except for a slightly reduced LVEF (48%). Her son is now more than 5 years old, and regular physical examination showed normal growth and development.

## Discussion

KSS is a rare form of MD and is predominantly caused by mtDNA rearrangement. The pathogenic mtDNA changes mainly presented a 4.9 kb “common deletion” range from nucleotide positions 8,470 to 13,446, including several important functional genes, including MT-ATP8, MT-ATP6, MT-CO3, MT-ND3, MT-ND4 L, MT-ND4, and MT-ND5(4). mtDNA deletion usually represents *de novo* mutational events occurring either in an oocyte or in the early stage of embryonic development. Thus, the majority of cases with KSS are sporadic, while only 4% of KSS cases can acquire this genetic abnormality by maternal inheritance (1). The clinical diagnosis of KSS depends on a classic triad (including onset before age 20, PEO, and pigmentary retinopathy) and at least one of the following comorbidities: cardiac conduction defects, cerebellar ataxia, and cerebrospinal fluid protein>100 mg/dL (5). Other clinical features, such as endocrine disorders, dysphagia, ptosis, muscle weakness, fatigue, and hearing loss, can also contribute to the diagnosis. The wide spectrum and different severities of clinical manifestations present in individuals with KSS are mainly due to the variable proportions of mtDNA deletions and their tissue distribution (6). Even with a similar mtDNA deletion, the clinical symptoms among those populations can be distinct. In the present patient, the diagnosis of KSS was based on the classic clinical manifestations (onset at the age of 14, PEO, retinal pigmentary changes) and concomitant cardiac conduction defects (RBBB and LAFB presented in ECG) and then confirmed by a typical “common deletion” detected by the genetic analysis of muscle biopsy. There was no family history of a similar condition. Genetic analysis of her parents showed no abnormal findings. Thus, we can confirm the diagnosis of the current case with sporadic KSS. However, whether her child can inherit such mtDNA deletions by a mode of maternal inheritance remains unclear and requires further follow-up.

Cardiac involvement, as a major prognostic factor of KSS, can take many forms, namely, cardiac conduction abnormalities and cardiomyopathy. As a part of the diagnostic criteria, cardiac conduction disorders occur in 84% of KSS patients (7). The most frequent ECG abnormality of KSS is represented by intraventricular and atrioventricular (AV) conduction delay (8). In KSS with fascicular blocks, the progression to a fatal completed AV block can usually be rapid and unpredictable, either in cases with or without symptoms. The limited literature shows that 11–20% of KSSs suffer SCD (3, 9), much higher than the estimated 0.1 0.2% in the general population (10). Thus, current ACCF/AHA/HRS guidelines recommend the implantation of a permanent pacemaker (PPM) for cases of KSS with third-degree and advanced second-degree AV block, even in asymptomatic cases (11). Cardiomyopathy, one of the most common clinical manifestations in KSS with cardiac involvement, may be hypertrophic or dilated. Myocardial involvement often means an increased risk of mortality. Heart failure (HF) is the leading cause of death among this group (2). Echocardiography showing wall motion abnormalities, which occur at later stages of myocardial involvement, may contribute to the diagnosis of cardiomyopathy. CMR with a typical pattern of diffuse intramural LGE in the left ventricular inferolateral walls may be useful in identifying early-stage myocardial damage in patients before abnormalities become apparent on echocardiography (12). Even if no functional abnormalities were found on echocardiography, the present case demonstrated cardiac involvement based on typical changes both on ECG and CMR before pregnancy that further developed into postpartum HF. Notwithstanding the remission of postpartum AHF by multidisciplinary therapy, the EF of this current case has not returned to normal. Considering the high mortality and risk of recurrence, subsequent pregnancy in such patients in whom EF does not normalize (>50–55%) should be discouraged (13). Even with a normalized EF, prepregnancy counseling is necessary.

Several retrospective studies of pregnancies in MD found that pregnancies are well tolerated, for the most part, and complications are very similar to those in the general population except for a higher incidence of early pregnancy loss, gestational diabetes mellitus, intrauterine growth retardation, and postpartum hemorrhage (14, 15). However, reports of pregnancy and delivery of KSS with cardiac involvement are rare, and it is yet unclear how pregnancy and delivery may affect the clinical course of KSS with cardiac involvement. Pregnancy is a high energy and circulation demand state that requires more workload on the heart to maintain normal physiological adaptation. Uterine contractions, painful stimuli, anxiety, fatigue, hemorrhaging, and anesthesia may induce additional stress and cardiovascular burden in the course of delivery. The postpartum period may precipitate the progression of myocardial dysfunction, particularly characterized by significant haemodynamic changes and fluid shifts in the first 24–48 h after delivery (13). In theory, KSS with cardiac involvement is more susceptible to adverse cardiac events since it is characterized by an impaired baseline of myocardial energy metabolism and decreased reserve capabilities. However, it is difficult to derive conclusions from a single case study, and further large and multicentric studies are essential to verify our conclusions.

In conclusion, to assess the pregnancy-related risk in female patients with KSS who are pregnant or wish to become pregnant, a detailed imaging assessment should be performed to identify the presence and degree of cardiac involvement. Further studies are required in KSS with cardiac involvement to explore optimal methods to predict the potential risk of adverse cardiac prognosis during pregnancy.

## Patient Perspective

The patient has no doubts about our treatment. She hopes this report will incentivize more investigations to elucidate the pathophysiological changes of KSS during the course of pregnancy and delivery, especially among cases with cardiac involvement. She thinks this rare but high-risk pregnancy group should get more attention.

## Author Contributions

All authors listed have made a substantial, direct, and intellectual contribution to the work and approved it for publication.

## Conflict of Interest

The authors declare that the research was conducted in the absence of any commercial or financial relationships that could be construed as a potential conflict of interest.

## Publisher's Note

All claims expressed in this article are solely those of the authors and do not necessarily represent those of their affiliated organizations, or those of the publisher, the editors and the reviewers. Any product that may be evaluated in this article, or claim that may be made by its manufacturer, is not guaranteed or endorsed by the publisher.
